# Improved efficacy and safety of zanubrutinib versus ibrutinib in patients with relapsed/refractory chronic lymphocytic leukemia (R/R CLL) in China: a subgroup of ALPINE

**DOI:** 10.1007/s00277-024-05823-8

**Published:** 2024-06-18

**Authors:** Keshu Zhou, Tingyu Wang, Ling Pan, Wei Xu, Jie Jin, Wei Zhang, Yu Hu, Jianda Hu, Ru Feng, Ping Li, Zhougang Liu, Peng Liu, Hongmei Jing, Sujun Gao, Huilai Zhang, Kang Yu, Zhao Wang, Xiongpeng Zhu, Zimin Sun, Fei Li, Dongmei Yan, Jianyu Weng, Lina Fu, Liping Wang, Tommi Salmi, Kenneth Wu, Lugui Qiu

**Affiliations:** 1 Affiliated Cancer Hospital of Zhengzhou University, Henan Cancer Hospital, Zhengzhou, China; 2 National Clinical Research Center for Hematological Disorders, State Key Laboratory of Experimental Hematology, Institute of Hematology and Blood Diseases Hospital, Chinese Academy of Medical Sciences and Peking Union Medical College, Tianjin, China; 3 Tianjin Institutes of Health Science, Tianjin, China; 4 West China Hospital, Sichuan University, Sichuan, China; 5 Jiangsu Province Hospital, Zhejiang, China; 6 The First Hospital of Zhejiang Province, Zhejiang, China; 7 Peking Union Medical College Hospital, Beijing, China; 8 Union Hospital of Tongji Medical College, Wuhan, China; 9 Fujian Medical University Union Hospital, Fuzhou, China; 10 Nanfang Hospital of Southern Medical University, Guangzhou, China; 11 Tongji Hospital of Tongji University, Wuhan, China; 12 Shengjing Hospital of China Medical University, Shenyang, China; 13 Zhongshan Hospital of Fudan University, Shanghai, China; 14 Peking University Third Hospital, Beijing, China; 15 The First Hospital of Jilin University, Changchun, China; 16 Tianjin Medical University Cancer Institute & Hospital, Tianjin, China; 17 The First Affiliated Hospital of Wenzhou Medical University, Zhejiang, China; 18 Beijing Friendship Hospital, Beijing, China; 19 Quanzhou First Hospital of Fujian Province, Quanzhou, China; 20 Anhui Provincial Hospital, Hefei, China; 21 The First Affiliated Hospital of Nanchang University, Nanchang, China; 22 The Affiliated Hospital of Xuzhou Medical University, Jiangsu, China; 23 Guangdong Provincial People’s Hospital, Guangzhou, China; 24 BeiGene, Ltd, Beijing, China; 25 BeiGene International GmbH, Basel, Switzerland; 26 BeiGene USA, Inc, San Mateo, CA USA

**Keywords:** Zanubrutinib, Ibrutinib, Chronic lymphocytic leukemia, Small lymphocytic lymphoma, Chinese population

## Abstract

Chronic lymphocytic leukemia/small lymphocytic lymphoma (CLL/SLL) has different epidemiology in Chinese vs. Western patients, but there are few studies of CLL/SLL in large populations of Chinese patients. ALPINE is a global phase 3 trial investigating Bruton tyrosine kinase inhibitors zanubrutinib vs. ibrutinib to treat relapsed/refractory (R/R) CLL/SLL. Here we report results from the subgroup of Chinese patients. Adults with R/R CLL/SLL were randomized 1:1 to receive zanubrutinib (160 mg twice-daily) or ibrutinib (420 mg once-daily) until disease progression or unacceptable toxicity. Endpoints included overall response rate (ORR), progression-free survival (PFS), overall survival (OS), and safety. Data were analyzed descriptively. Ninety patients were randomized in China (zanubrutinib, *n* = 47; ibrutinib, *n* = 43). Baseline characteristics were balanced between groups, with fewer male patients in the zanubrutinib vs. ibrutinib group (55.3% vs. 69.8%). Median age was 60.5 years, 11% had del(17p) mutation, and 32% had tumor protein 53 (*TP53*) mutation. With median 25.3 months follow-up, ORR was 80.9% with zanubrutinib vs. 72.1% with ibrutinib. PFS was improved with zanubrutinib vs. ibrutinib (HR = 0.34 [95% CI, 0.15, 0.77]), and the HR for OS was 0.45 (95% CI, 0.14, 1.50). Rates of Grade ≥ 3 treatment-emergent adverse events (TEAEs; 64.4% vs. 72.1%), AEs leading to discontinuation (6.4% vs. 14.0%), and serious TEAEs (35.6% vs. 51.2%) were lower with zanubrutinib vs. ibrutinib. Zanubrutinib demonstrated improved ORR, PFS, and OS vs. ibrutinib and a more favorable safety profile in patients with R/R CLL/SLL in China. These results are consistent with the full global population of ALPINE. ClinicalTrials.gov: NCT03734016, registered November 7, 2018.

**Supplementary Information:**

The online version contains supplementary material available at 10.1007/s00277-024-05823-8.

## Introduction

Chronic lymphocytic leukemia (CLL) is the most common type of leukemia in the Western hemisphere, with an age-adjusted incidence of 1.28 per 100,000 people worldwide in 2019 [1]. Small lymphocytic lymphoma (SLL) is a different manifestation of the same disease [2]; throughout this manuscript, CLL/SLL will simply be referred to as CLL. There is heterogeneity in the epidemiology of CLL between Western countries and China. For example, the age-adjusted incidence rate of CLL in China is 0.2–0.6 per 100,000, substantially lower than that in patients of European descent [3]. Furthermore, although the incidence ratio of men to women with CLL is similar between the United States and China (1.9:1 and 1.8:1, respectively), the median age at diagnosis in the United States is 70 years compared with 58–62 years in China [4, 5]. Among patients diagnosed with CLL between 2010 and 2018, the overall survival (OS) in Chinese patients was significantly prolonged compared with that in patients in the United States (*P* = .047), which may be a result of the younger age at diagnosis in Chinese patients [4]. Additionally, while newly diagnosed Chinese patients with CLL and Western patients have similar frequencies of del(17p) (7.8% [6] vs. 5–8% [5], respectively) and *TP53* mutations (8.2% [6] vs. 4–8% [7]), the frequency of unmutated immunoglobulin heavy chain variable region in untreated CLL is lower in Chinese patients (31% [6] vs. 48% [8]). In an analysis of Chinese vs. Western patients with CLL, *MYD88* mutations (12.5% vs. 3.0–3.6%) and *KMT2D* mutations (7.9% vs. 0.7–1.1%) were more frequent in the Chinese population, and *MYD88* mutations were significantly more common in newly diagnosed vs. relapsed CLL [9]. The frequency of *TP53* mutations in relapsed/refractory (R/R) CLL is similar in Chinese vs. Western patients (16–43% [10] vs. 30–40% [7]). Collectively, these factors can influence disease prognosis [5, 9, 11]. Despite the differences in disease presentation in Chinese and Western patients, no population-based studies of CLL have been conducted in mainland China, and studies in large populations of Chinese patients with CLL are rare [3].

For many years, the standard of care for R/R CLL in China included rituximab, alkylating agents, and fludarabine [3]. In 2013, the Bruton tyrosine kinase (BTK) inhibitor ibrutinib was approved for the treatment of R/R CLL in China [3]. Since its approval, ibrutinib has revolutionized the treatment of CLL, despite its association with treatment resistance and increased risk for cardiovascular adverse events (AEs) such as atrial fibrillation, cardiac failure, bleeding, and hypertension [5].

Zanubrutinib, a next-generation, irreversible, potent, selective BTK inhibitor designed to maximize BTK occupancy and minimize off-target inhibition [12, 13], was approved for the treatment of CLL in China in 2020 [3]. The global phase 3 ALPINE trial (NCT03734016) evaluated the efficacy and safety of zanubrutinib vs. ibrutinib in patients with R/R CLL or SLL [13, 14]. The results showed that zanubrutinib treatment had a superior overall response rate (ORR) [14], significantly longer progression-free survival (PFS), and fewer AEs, including cardiac AEs, compared with ibrutinib [13]. Here, we report results from the ALPINE trial in the subgroup of patients enrolled in China.

## Methods

### Study design and population

ALPINE (NCT03734016) is a global, phase 3, open-label, randomized study investigating the efficacy and safety of zanubrutinib vs. ibrutinib in patients with R/R CLL/SLL. The trial design is shown in Supplementary Fig. 1, and the methodological details have been reported previously [13]. Male and female patients were eligible to participate in the study if they were ≥ 18 years old, had a confirmed diagnosis of CLL or SLL requiring treatment per the International Workshop on CLL criteria [15], experienced relapse or had disease refractory to ≥ 1 prior systemic therapy for CLL/SLL, and had an Eastern Cooperative Oncology Group performance status of 0–2. Patients with known prolymphocytic leukemia or a history of Richter’s transformation, clinically significant cardiovascular disease, prior malignancy in the past 3 years, history of severe bleeding disorder or stroke, severe pulmonary disease, or prior treatment with a BTK inhibitor were excluded.

Patients were randomized 1:1 to receive either zanubrutinib 160 mg orally twice daily or ibrutinib 420 mg orally once daily until disease progression or unacceptable toxicity. Randomization was stratified by age (< 65 years vs. ≥ 65 years), geographic region (China vs. non-China), refractory status (yes or no), and del(17p)/*TP53* mutation status (present or absent). This analysis presents data for patients enrolled in China.

### Assessments

In this subgroup analysis, ORR assessed by blinded independent review committee (IRC) and by the investigator (INV) was evaluated. ORR was defined as a complete response or a complete response with incomplete bone marrow recovery, a nodular partial response, or a partial response. Other key assessments included PFS, duration of response (DoR), and rate of partial response with lymphocytosis or better by IRC and INV; time to treatment failure; OS; and safety. Efficacy was assessed via symptoms, physical examination, computerized tomography imaging, and laboratory tests; assessment of CLL was according to International Workshop on CLL criteria [15] with the addition of treatment-related lymphocytosis [16], and SLL was assessed according to Lugano classification [17]. AEs were assessed and graded based on the National Cancer Institute Common Terminology Criteria for Adverse Events v4.03. AEs of special interest (AESI) were prespecified pooled categories (Supplementary Methods).

### Statistical analysis

Demographic and baseline characteristics and efficacy were summarized for all Chinese patients randomized. Safety analyses were summarized for all Chinese patients who received ≥ 1 dose of study drug. All analyses were descriptive, and no formal hypothesis testing was performed; therefore, no statements of statistical significance can be made.

### Compliance with ethical standards

This study was conducted in accordance with the Declaration of Helsinki and the International Conference on Harmonization Guidelines for Good Clinical Practice. Written informed consent was obtained from each patient, and institutional review board approval was obtained at each study site.

### Data sharing statement

BeiGene voluntarily shares anonymous data on completed studies responsibly and provides qualified scientific and medical researchers access to anonymous data and supporting clinical trial documentation for clinical trials in dossiers for medicines and indications after submission and approval in the United States, China, and Europe. Clinical trials supporting subsequent local approvals, new indications, or combination products are eligible for sharing once corresponding regulatory approvals are achieved. BeiGene shares data only when permitted by applicable data privacy and security laws and regulations. In addition, data can only be shared when it is feasible to do so without compromising the privacy of study participants. Qualified researchers may submit data requests/research proposals for BeiGene review and consideration through BeiGene’s Clinical Trial Webpage at https://www.beigene.com/our-science-and-medicines/our-clinical-trials/.

## Results

### Patients

**Fig. 1:**
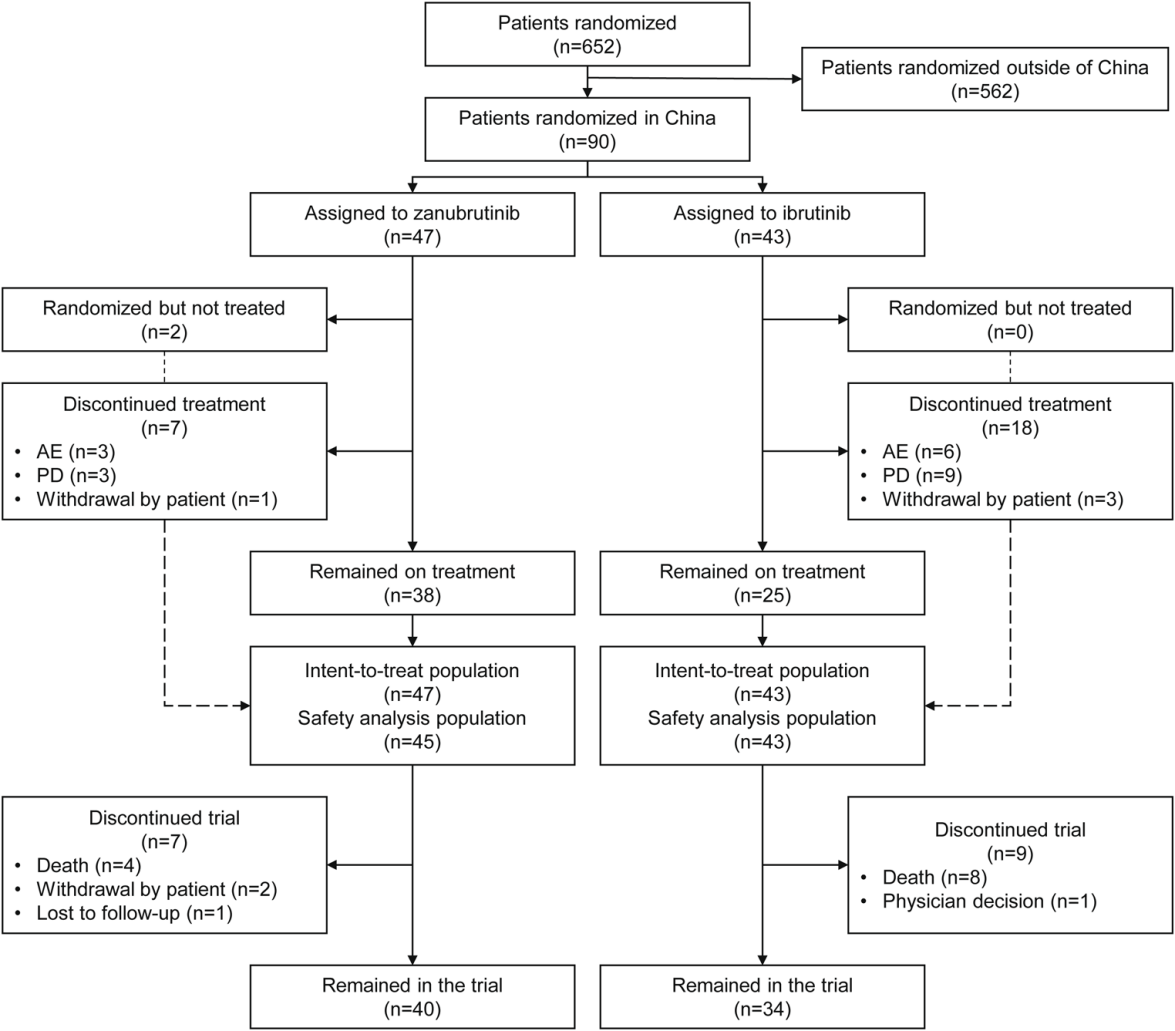
Patient disposition. AE, adverse event; PD, progressive disease

A total of 652 patients were randomized by the cutoff date of August 8, 2022, 90 of whom were from China. Forty-seven were randomized to receive zanubrutinib and 43 to receive ibrutinib (Fig. 1). The demographic and clinical characteristics in both treatment groups were consistent at baseline; however, there were fewer male patients in the zanubrutinib group than in the ibrutinib group (55.3% vs. 69.8%, respectively) (Table 1). The median age was 60.5 years (range, 35–82), 41.1% of patients had bulky disease (i.e., a tumor that was ≥ 5 cm diameter), 11.1% had del(17p), and 32.2% had *TP53* mutation. The median number of prior lines of therapy was 1 (range, 1–12), with most patients (85 [94.4%]) having received an alkylating agent, excluding bendamustine. The representativeness of study participants compared with the Chinese and world populations can be found in Supplementary Table 1.

**Table 1:** Demographics and baseline characteristics of patients in the Chinese subgroup (ITT)

	Zanubrutinib (*n* = 47)	Ibrutinib (*n* = 43)	Total (*N* = 90)
Age, median (range), years	60 (35–82)	61 (35–80)	60.5 (35–82)
< 65 years, n (%)	28 (59.6)	27 (62.8)	55 (61.1)
≥ 65 to < 75 years, n (%)	17 (36.2)	15 (34.9)	32 (35.6)
≥ 75 years, n (%)	2 (4.3)	1 (2.3)	3 (3.3)
Male, n (%)	26 (55.3)	30 (69.8)	56 (62.2)
ECOG PS, n (%)			
0–1	46 (97.9)	42 (97.7)	88 (97.8)
2	1 (2.1)	1 (2.3)	2 (2.2)
Mutational status, n (%)			
del(17p) present	6 (12.8)	4 (9.3)	10 (11.1)
del(11q) present	11 (23.4)	11 (25.6)	22 (24.4)
*TP53* mutated	15 (31.9)	14 (32.6)	29 (32.2)
del(17p) and/or *TP53* mutation	16 (34.0)	14 (32.6)	30 (33.3)
*IGHV* unmutated	28 (59.6)	27 (62.8)	55 (61.1)
Bulky disease, n (%)			
Any target lesion longest diameter ≥ 5 cm	19 (40.4)	18 (41.9)	37 (41.1)
Any target lesion longest diameter ≥ 10 cm	2 (4.3)	3 (7.0)	5 (5.6)
β2 microglobulin, n (%)			
≤ 3.5 mg/L	17 (36.2)	11 (25.6)	28 (31.1)
> 3.5 mg/L	30 (63.8)	30 (69.8)	60 (66.7)
Data missing	0	2 (4.7)	2 (2.2)
Lactate dehydrogenase level, median (range), U/L	217 (143–1234)	216 (132–600)	216.5 (132–1234)
Disease stage, n (%)			
Binet stage A or B or Ann Arbor stage I or II	21 (44.7)	19 (44.2)	40 (44.4)
Binet stage C or Ann Arbor stage III or IV	26 (55.3)	24 (55.8)	50 (55.6)
Previous systemic therapy			
Lines of therapy, median (range), n	1 (1–4)	1 (1–12)	1 (1–12)
1, n (%)	28 (59.6)	22 (51.2)	50 (55.6)
2, n (%)	13 (27.7)	11 (25.6)	24 (26.7)
3, n (%)	3 (6.4)	4 (9.3)	7 (7.8)
> 3, n (%)	3 (6.4)	6 (14.0)	9 (10.0)
Types of therapy, n (%)			
Anti-CD20 antibody	21 (44.7)	17 (39.5)	38 (42.2)
Alkylating agent, excluding bendamustine	45 (95.7)	40 (93.0)	85 (94.4)
Chemoimmunotherapy	21 (44.7)	15 (34.9)	36 (40.0)
Purine analogue	27 (57.4)	27 (62.8)	54 (60.0)
Bendamustine	1 (2.1)	1 (2.3)	2 (2.2)
BCL2 inhibitor	0	1 (2.3)	1 (1.1)
Immunomodulatory drug	3 (6.4)	0	3 (3.3)
Alemtuzumab	1 (2.1)	0	1 (1.1)

SI conversion factor for lactate dehydrogenase: U/L to µkat/L, multiply by 0.0167

BCL2, B-cell lymphoma 2; CD20, cluster of differentiation 20; del(11q), deletion in chromosome 11q; del(17p), deletion in chromosome 17p; ECOG PS, Eastern Cooperative Oncology Group performance status; *IGHV*, immunoglobulin heavy chain variable region; ITT, intent-to-treat; *TP53*, tumor protein 53; U, units

### Overall response

**Fig. 2:**
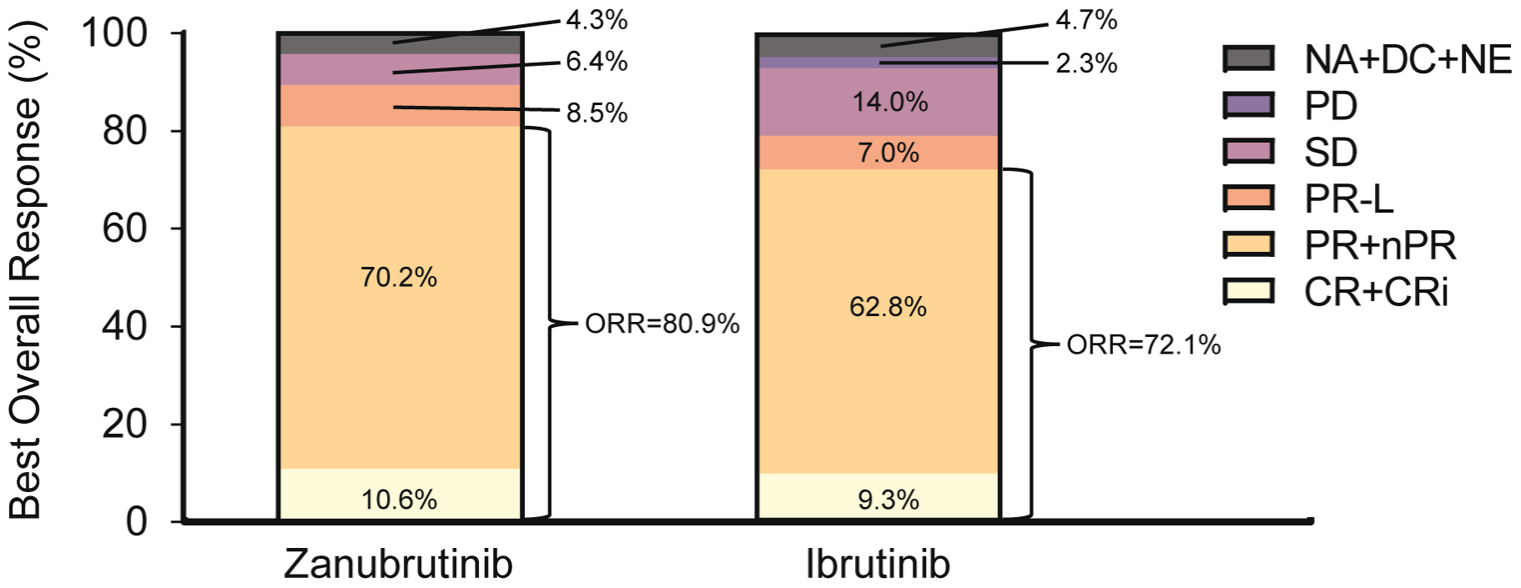
Best overall response by INV in the Chinese subgroup (ITT). CR, complete response; CRi, complete response with incomplete bone marrow recovery; DC, discontinued prior to first assessment; INV, investigator assessment; ITT, intent-to-treat; NA, not assessed; NE, not evaluable; nPR, nodular partial response; ORR, overall response rate; PD, progressive disease; PR, partial response; PR-L, partial response with lymphocytosis; SD, stable disease

With an overall median study follow-up of 25.3 months (range, 0.1–40.4), the ORR assessed by INV was higher in the zanubrutinib-treated group than in the ibrutinib group (80.9% vs. 72.1%, respectively) (Fig. 2). As assessed by IRC, the ORR was 87.2% with zanubrutinib and 76.7% with ibrutinib (Supplementary Table 2). A higher percentage of patients had partial response with lymphocytosis or better by INV in the zanubrutinib group (89.4%) vs. the ibrutinib group (79.1%), which is consistent with the analysis by IRC (89.4% vs. 83.7%, respectively). For DoR as assessed by INV, responders in the zanubrutinib group had an 18-month event-free rate of 91.3% (95% CI, 75.5, 97.1) compared with 69.2% (95% CI, 49.1, 82.7) in the ibrutinib group. These values were 94.7% (95% CI, 80.3, 98.6) vs. 77.5% (95% CI, 58.3, 88.6), respectively, as assessed by IRC (Supplementary Table 3).

### Progression-free survival

**Fig. 3:**
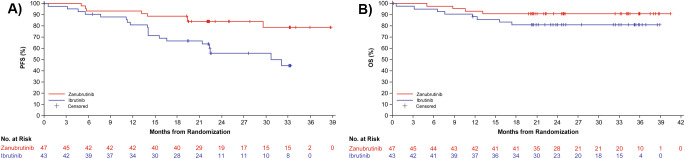
PFS by INV (**a**) and OS (**b**) in the Chinese subgroup (ITT). INV, investigator assessment; ITT, intent-to-treat; OS, overall survival; PFS, progression-free survival

With a median follow-up of 22.6 months for the PFS endpoint, PFS by INV was improved with zanubrutinib vs. ibrutinib (HR, 0.34 [95% CI, 0.15, 0.77]) (Fig. 3A). There were 6 (12.8%) vs. 14 (32.6%) events of progressive disease (PD) and 2 (4.3%) vs. 5 (11.6%) deaths without PD in the zanubrutinib vs. ibrutinib groups, respectively. Zanubrutinib also showed improved PFS compared with ibrutinib when assessed by IRC (Supplementary Fig. 2). The PFS rate by INV at 18 months was 88.9% (95% CI, 75.3, 95.2) with zanubrutinib vs. 66.8% (95% CI, 50.5, 78.8) with ibrutinib; at 24 months it was 84.1% (95% CI, 69.5, 92.1) vs. 55.8% (95% CI, 37.7, 70.5). Median PFS by INV was not reached in the zanubrutinib group and was 32.1 months (95% CI, 21.4, not evaluable) in the ibrutinib group. At 24 months, 88.1% (95% CI, 73.4, 94.9) in the zanubrutinib group vs. 62.0% (95% CI, 45.6, 74.8) in the ibrutinib group were free from treatment failure (Supplementary Fig. 3).

In the high-risk subgroup of patients with del(17p) or *TP53* mutation, PFS also favored the zanubrutinib group over the ibrutinib group as assessed by INV (HR, 0.49 [95% CI, 0.14, 1.67]), and IRC (HR, 0.51 [95% CI, 0.12, 2.13]). There were 3 (18.8%) vs. 5 (35.7%) instances of PD and 2 (12.5%) vs. 2 (14.3%) deaths without PD in the zanubrutinib vs. ibrutinib groups, respectively. The PFS rate by INV at 18 months was 73.3% (95% CI, 43.6, 89.1) with zanubrutinib and 50.0% (95% CI, 22.9, 72.2) with ibrutinib.

### Overall survival

A total of 4 (8.5%) patients in the zanubrutinib group and 8 (18.6%) in the ibrutinib group died. The HR for OS was 0.45 (95% CI, 0.14, 1.50) with zanubrutinib vs. ibrutinib. Median OS had not been reached in either group (Fig. 3B). The OS rate at 24 months was 91.1% (95% CI, 78.0, 96.6) with zanubrutinib and 81.2% (95% CI, 65.9, 90.1) with ibrutinib.

### Safety

The median duration of treatment in the zanubrutinib group was 25.5 months (range, 3.6, 39.2) vs. 23.7 months (range, 0.1, 38.5) in the ibrutinib group. Table 2 summarizes the non-hematologic treatment-emergent AEs (TEAEs) that occurred in ≥ 15% of patients in either treatment group. Upper respiratory tract infection (URTI) occurred in 35.6% vs. 30.2% in the zanubrutinib group vs. ibrutinib group, respectively. Hematologic TEAEs are reported as pooled terms among the AESI below.

**Table 2:** Safety overview in the Chinese subgroup (safety analysis set)

TEAEs, *n* (%)	Zanubrutinib (*n* = 45)	Ibrutinib (*n* = 43)
≥ 1 TEAE	44 (97.8)	42 (97.7)
Grade ≥ 3 TEAEs	29 (64.4)	31 (72.1)
Serious TEAEs	16 (35.6)	22 (51.2)
Leading to dose modification	10 (22.2)	13 (30.2)
TEAEs leading to discontinuation	3 (6.7)	6 (14.0)
TEAEs leading to death	2 (4.4)	3 (7.0)
Non-hematologic TEAEs occurring in ≥ 15% of patients in either arm		
URTI	16 (35.6)	13 (30.2)
Pneumonia	11 (24.4)	13 (30.2)
Hyperuricemia	9 (20.0)	10 (23.3)
Rash	9 (20.0)	12 (27.9)
Hypokalemia	8 (17.8)	5 (11.6)
Urinary tract infection	7 (15.6)	3 (7.0)
Blood creatinine increased	7 (15.6)	4 (9.3)
Diarrhea	6 (13.3)	7 (16.3)
Pyrexia	4 (8.9)	7 (16.3)

TEAE, treatment-emergent adverse event; URTI, upper respiratory tract infection

Grade ≥ 3 TEAEs occurred in fewer patients in the zanubrutinib group vs. the ibrutinib group (29 [64.4%] vs. 31 [72.1%]). Non-hematologic Grade ≥ 3 TEAEs that occurred in ≥ 10% of patients in either arm were pneumonia (13.3% vs. 18.6%) and URTI (11.1% vs. 7.0%) (Supplementary Table 4). Serious TEAEs also occurred less frequently with zanubrutinib vs. ibrutinib (16 [35.6%] vs. 22 [51.2%]). In the zanubrutinib group, 2 patients (4.4%) had TEAEs leading to death vs. 3 (7.0%) in the ibrutinib group (Supplementary Table 5). The most common fatal TEAEs were infections and infestations, which occurred in 2 patients (4.4%) in the zanubrutinib group and 1 patient (2.3%) in the ibrutinib group.

In the intent-to-treat population, the treatment discontinuation rate was lower with zanubrutinib (14.9%) vs. ibrutinib (41.9%). Most treatment discontinuation was due to PD (6.4% vs. 20.9%, respectively) and AEs, and fewer AEs led to discontinuation in the zanubrutinib group than in the ibrutinib group (3 [6.4%] vs. 6 [14.0%]). No COVID-19–related TEAEs occurred.

Table 3 shows a summary of AESI. Anemia (zanubrutinib vs. ibrutinib, 31.1% vs. 39.5%), atrial fibrillation/flutter (0% vs. 4.7%), infections of any kind (77.8% vs. 79.1%), neutropenia (42.2% vs. 51.2%), skin cancer (0% vs. 2.3%), and thrombocytopenia (28.9% vs. 34.9%) all occurred less frequently in the zanubrutinib vs. the ibrutinib group. No Grade ≥ 3 atrial fibrillation/flutter or hemorrhage occurred in either group. Opportunistic infections of any grade occurred in 1 patient (2.2%) in the zanubrutinib group vs. 3 (7.0%) in the ibrutinib group (Supplementary Table 6).

**Table 3:** AESI in the Chinese subgroup (safety analysis set)

AESI, *n* (%)^a^	Any grade		Grade ≥ 3	
	Zanubrutinib (*n* = 45)	Ibrutinib (*n* = 43)	Zanubrutinib (*n* = 45)	Ibrutinib (*n* = 43)
Any AESI	40 (88.9)	41 (95.3)	24 (53.3)	27 (62.8)
Anemia	14 (31.1)	17 (39.5)	1 (2.2)	1 (2.3)
Atrial fibrillation/flutter	0	2 (4.7)	0	0
Hemorrhage	22 (48.9)	15 (34.9)	0	0
Major hemorrhage	0	0	0	0
Hypertension	7 (15.6)	6 (14.0)	2 (4.4)	2 (4.7)
Infections	35 (77.8)	34 (79.1)	14 (31.1)	16 (37.2)
Opportunistic infections	1 (2.2)	3 (7.0)	1 (2.2)	1 (2.3)
Neutropenia	19 (42.2)	22 (51.2)	11 (24.4)	13 (30.2)
Second primary malignancies	0	5 (11.6)	0	5 (11.6)
Skin cancers	0	1 (2.3)	0	1 (2.3)
Thrombocytopenia	13 (28.9)	15 (34.9)	2 (4.4)	5 (11.6)

^a^Specific related Medical Dictionary for Regulatory Activities preferred terms were pooled for each AESI category and summarized; a summary of AESI categories and search criteria can be found in the Supplemental Methods

AESI, adverse events of special interest

## Discussion

The ALPINE study was the first head-to-head comparison of zanubrutinib vs. ibrutinib for treatment of R/R CLL/SLL [13]. While differences in demographic and baseline characteristics were evident between Chinese patients and the intent-to-treat population of ALPINE, efficacy and safety findings were largely consistent between the 2 groups.

In comparison with the full population of the ALPINE study, the demographic and disease characteristics in the Chinese population had some notable differences. Chinese patients who participated in ALPINE were younger (median age, 60.5 years) than those in the overall population (median age, 67 years) [13], which is congruent with published data [4, 5]. This is relevant as younger patients with CLL tend to have longer OS than their older counterparts. However, relapse rates are higher in younger patients [4], and the recommended treatment for younger patients differs from that for older patients [2]. Additionally, a higher proportion of Chinese vs. full-population patients had a higher disease stage (55.6% vs. 42.9% with Binet stage C or Ann Arbor stage III or IV), which aligns with published data on CLL in Chinese vs. European populations [3]. Chinese patients also had a higher presence of del(17p) or *TP53* mutations (33.3%) compared with the full population (23.0%), which is important because both factors can influence response to therapy and prognosis [5]. Prior treatment with alkylating agents may have contributed to the greater percentage of *TP53* mutations in the Chinese population [18], as a higher percentage of patients in the Chinese population (94.4%) were previously treated with alkylating agents (excluding bendamustine) vs. the full population (81.6%) [13]. Other previous treatment regimens also differed between the populations, including a lower proportion of Chinese patients previously treated with anti-CD20 antibodies (42.2%) and bendamustine (2.2%) compared with the full population (83.3% and 27.3%, respectively).

Despite differences in demographic and disease characteristics between the Chinese population subgroup and the overall population of ALPINE, efficacy results consistently favored zanubrutinib over ibrutinib in both populations. The median follow-up of 25.3 months in the Chinese population was shorter than that in the full population (29.6 months [13]) because patients enrolled in China joined the ALPINE study later than those in the rest of the world. Nonetheless, in both populations, the ORR was higher in the zanubrutinib vs. ibrutinib group (ORR by INV: Chinese, 80.9% vs. 72.1%; full, 83.5% vs. 74.2%). PFS rates by both INV and IRC were also higher in the zanubrutinib group vs. the ibrutinib group in both the Chinese population and the overall population. The PFS rate with zanubrutinib vs. ibrutinib at 24 months in the Chinese population was 84.1% vs. 55.8%, while in the full population, it was 78.4% vs. 65.9%. Additionally, in the subset of patients with del(17p)/*TP53* mutation, PFS was favorable in the zanubrutinib group for both the Chinese (HR [INV], 0.49) and the full (HR [INV], 0.53) populations. Other endpoints such as OS, DoR, and time to treatment failure were also improved with zanubrutinib vs. ibrutinib in the Chinese population.

Zanubrutinib was well tolerated and had an improved safety profile over ibrutinib in both the full study population and patients enrolled in China. The rate of AEs leading to treatment discontinuation was lower with zanubrutinib vs. ibrutinib in the Chinese subgroup. While both the Chinese and the full populations showed lower rates of atrial fibrillation/flutter in the zanubrutinib group vs. the ibrutinib group, there were also fewer instances of both atrial fibrillation/flutter and hypertension in the Chinese vs. the full population. There were no instances of major hemorrhage in the Chinese population vs. 4% in both zanubrutinib and ibrutinib arms in the full population. Compared with the full population, a higher proportion of patients in the Chinese subgroup experienced adverse events of infections (full population: zanubrutinib, 71.3% and ibrutinib, 73.1% [13]; Chinese subgroup: zanubrutinib, 77.8% and ibrutinib, 79.1%). No COVID-19–related TEAEs were reported in the Chinese subgroup as of the data cutoff date. In the full population, 93 patients (28.7%) receiving zanubrutinib and 70 patients (21.6%) receiving ibrutinib reported any COVID-19–related TEAE including COVID-19, COVID-19 pneumonia, post-acute COVID-19 syndrome, and suspected COVID-19 [13].

No cardiac disorders leading to death occurred in either group in the Chinese population. The low number of cardiac events in Chinese patients was likely due to the small number of patients in the subgroup but may also be related to patients being younger and having a favorable cardiac risk profile at baseline, such as low body mass index, low frequency of hypertension, and low frequency of diabetes [19]. There were also no second primary malignancies in the zanubrutinib group vs. 5 (11.6%) in the ibrutinib group in the Chinese population. Other notable differences included a lower incidence of skin cancer and higher incidences of URTI, neutropenia, and thrombocytopenia in the Chinese vs. full population, regardless of treatment group. While the safety profile in the Chinese subgroup may have been slightly different than that in the global population, perhaps attributable to differences in baseline characteristics and disease background, the overall results suggest that zanubrutinib was more tolerable than ibrutinib in the Chinese population, consistent with findings in the rest of the world.

Limitations to the interpretation of this analysis include a Chinese subgroup size that was not powered to draw statistical conclusions and the need for more time to fully evaluate the OS and PFS findings.

## Conclusions

Efficacy and safety results favoring zanubrutinib over ibrutinib observed in patients enrolled in China were consistent with findings in the global population of the ALPINE study. In the Chinese subgroup, zanubrutinib continued to show improved PFS and ORR over ibrutinib, including in high-risk patients. A favorable safety profile of zanubrutinib vs. ibrutinib was also observed in patients from China, with lower rates of treatment discontinuation and serious AEs in patients treated with zanubrutinib.

## Electronic supplementary material

Below is the link to the electronic supplementary material.

Supplementary Material 1

## Data Availability

BeiGene voluntarily shares anonymous data on completed studies responsibly and provides qualified scientific and medical researchers access to anonymous data and supporting clinical trial documentation for clinical trials in dossiers for medicines and indications after submission and approval in the United States, China, and Europe. Clinical trials supporting subsequent local approvals, new indications, or combination products are eligible for sharing once corresponding regulatory approvals are achieved. BeiGene shares data only when permitted by applicable data privacy and security laws and regulations. In addition, data can only be shared when it is feasible to do so without compromising the privacy of study participants. Qualified researchers may submit data requests/research proposals for BeiGene review and consideration through BeiGene’s Clinical Trial Webpage at https://www.beigene.com/our-science-and-medicines/our-clinical-trials/.
