# Supplementary material for: Improved efficacy and safety of zanubrutinib versus ibrutinib in patients with relapsed/refractory chronic lymphocytic leukemia (R/R CLL) in China: a subgroup of ALPINE
Source: Ann Hematol. 2024 Jun 18;103(10):4183–91. doi: 10.1007/s00277-024-05823-8 (PMC11512864; doi:10.1007/s00277-024-05823-8)
Supplement: Supplementary file 1 — Supplementary Material 1 [file 277_2024_5823_MOESM1_ESM.pdf]

SUPPLEMENT

Improved efficacy and safety of zanubrutinib versus ibrutinib in patients with relapsed/refractory chronic lymphocytic leukemia (R/R CLL) in China: a subgroup of ALPINE

Authors: Keshu Zhou, Tingyu Wang, Ling Pan, Wei Xu, Jie Jin, Wei Zhang, Yu Hu, Jianda Hu, Ru Feng, Ping Li, Zhougang Liu, Peng Liu, Hongmei Jing, Sujun Gao, Huilai Zhang, Kang Yu, Zhao Wang, Xiongpeng Zhu, Zimin Sun, Fei Li, Dongmei Yan, Jianyu Weng, Lina Fu, Liping Wang, Tommi Salmi, Kenneth Wu, and Lugui Qiu

Corresponding Author:

Lugui Qiu

National Clinical Research Center for Hematological Disorders

State Key Laboratory of Experimental Hematology

Institute of Hematology and Blood Diseases Hospital

Chinese Academy of Medical Sciences and Peking Union Medical College

Tianjin Institutes of Health Science

Tianjin, China

Email: qiulg@ihcams.ac.cn

Journal:

Annals of Hematology

SUPPLEMENTARY METHODS

AESI Categories and Search Criteria

Category	Search Criteria
**Anemia**	Anemia PT, Hemoglobin decreased PT
**Atrial fibrillation/flutter**	Atrial fibrillation PT, Atrial flutter PT
**Hemorrhage**	Hemorrhage terms (excluding laboratory terms) (SMQ) Narrow
**Hypertension**	Hypertension (SMQ) Narrow
**Infection**	Infections: Infections and Infestations SOC
Opportunistic infections	Subcategory - Opportunistic infections: Opportunistic infections (SMQ) Narrow
**Major hemorrhage**	Major hemorrhage:<br>• Subdural hematoma PT<br>• Subdural hemorrhage PT<br>• All Hemorrhage PT if AE SOC is 'Nervous system disorders<br>• Serious or grade 3 and above Hemorrhage PT if AESOC is not 'Nervous system disorders
**Neutropenia**	Neutropenia PT, Neutrophil count decreased PT, Febrile neutropenia PT, Agranulocytosis PT, Neutropenic infection PT, Neutropenic sepsis PT
**Second primary malignancies**	Malignant Tumors (SMQ) Narrow
Skin cancers	Subcategory - skin cancers: skin malignant tumors (SMQ) Narrow
**Thrombocytopenia**	Thrombocytopenia PT, Platelet count decreased PT
**TLS**	Tumor lysis syndrome (SMQ) Narrow

AE, adverse events; AESI, adverse events of special interest; MedDRA, Medical Dictionary for Regulatory Activities; PT, preferred term; SMQ, standardized MedDRA query; SOC, system organ class; TLS, tumor lysis syndrome.

SUPPLEMENTARY TABLES

Supplementary Table 1. Representativeness of study participants

**Cancer type**	CLL/SLL
**Considerations related to:**	
Sex	CLL/SLL is more common in male than female patients, with a male-to-female incidence ratio of 1.9:1 in the United States [1] and 1.8:1 in China [2].
Age	The median age at diagnosis is 70 years in the United States [1] and 58-62 years in China [2].
Race and ethnicity	In the United States, CLL/SLL predominantly affects White patients, followed by Black, American Indian/Alaska Native, Hispanic, and Pacific Islander patients [1].
Geography	CLL/SLL predominantly affects Western patients. The incidence rate is lower in the Chinese population (0.2-0.6 per 100,000) [3] than in the worldwide population (1.3 per 100,000) [4].
Other considerations	Certain chromosomal abnormalities can influence disease prognosis in patients with CLL/SLL [5-7]. Patients with del(17p) or mutations of *TP53* tend to have worse outcomes than patients without [6,7]. *TP53* mutations occur in 30%-40% of patients with R/R CLL/SLL [8], while del(17p) occurs in approximately 7.8% of TN patients [9].
Overall representativeness of study	The analysis presented here is in patients with R/R CLL/SLL in China. Therefore, it is important to note that these patients do not represent the worldwide population of patients with CLL/SLL. In this study, 62%

	were male, the median age was 61 years (range, 35-82), 11.1% had del(17p), 32.2% had mutated *TP53*, and 61.1% had unmutated *IGHV*. Due to the nature of the analysis, 100% of patients were Asian.

CLL/SLL, chronic lymphocytic leukemia/small lymphocytic lymphoma; del(17p), deletion in chromosome 17p; *IGHV*, immunoglobulin heavy chain variable region; R/R, relapsed/refractory; TN, treatment naive; *TP53*, tumor protein 53.

Supplementary Table 2. Best responses by INV and IRC in the Chinese subgroup (ITT)

	ITT population	
	**Zanubrutinib (n=47)**	**Ibrutinib (n=43)**
**INV**		
**ORR, n (%) (95% CI)**	38 (80.9) (66.7, 90.9)	31 (72.1) (56.3, 84.7)
CR or CRi[a]	5 (10.6)	4 (9.3)
PR or nPR[b]	33 (70.2)	27 (62.8)
**PR-L**	4 (8.5)	3 (7.0)
**SD**	3 (6.4)	6 (14.0)
**PD**	0	1 (2.3)
**Discontinued prior to first assessment, NA or NE[c]**	2 (4.3)	2 (4.7)
**IRC**		
**ORR, n (%) (95% CI)**	41 (87.2) (74.3, 95.2)	33 (76.7) (61.4, 88.2)
CR or CRi[a]	4 (8.5)	4 (9.3)
PR or nPR[b]	37 (78.7)	29 (67.4)
**PR-L**	1 (2.1)	3 (7.0)
**SD**	2 (4.3)	4 (9.3)
**PD**	0	1 (2.3)
**Discontinued prior to first assessment, NA or NE[d]**	2 (4.3)	2 (4.7)

CI widths have not been adjusted for multiplicity and may not be used in place of hypothesis testing.

CI, confidence interval; CR, complete response; CRi, complete response with incomplete bone marrow recovery; INV, investigator assessment; IRC, independent review committee assessment; ITT, intent-to-treat; NA, not assessed; NE, not evaluable; nPR, nodular partial response; PD, progressive disease; PR, partial response; PR-L, partial response with lymphocytosis; ORR, overall response rate; SD, stable disease.

[a]No patients had CRi. [b]No patients had nPR. [c]No patients were NA or NE. [d]No patients were NA.

Supplementary Table 3. DoR by INV and IRC in the Chinese subgroup (ITT)

	**Zanubrutinib (n=47)**	**Ibrutinib (n=43)**
**INV**		
**Number of responders**	38	31
**Events, n (%)**	4 (10.5)	11 (35.5)
PD	3 (7.9)	11 (35.5)
Death	1 (2.6)	0
**DoR, median (95% CI), months**	NE (NE, NE)	25.1 (15.1, NE)
**18-month event-free rate (95% CI), %**	91.3 (75.5, 97.1)	69.2 (49.1, 82.7)
**IRC**		
**Number of responders**	41	33
**Events, n (%)**	2 (4.9)	10 (30.3)
PD	1 (2.4)	9 (27.3)
Death	1 (2.4)	1 (3.0)
**DoR, median (95% CI), months**	NE (NE, NE)	NE (19.4, NE)
**18-month event-free rate (95% CI), %**	94.7 (80.3, 98.6)	77.5 (58.3, 88.6)

CI widths have not been adjusted for multiplicity and may not be used in place of hypothesis testing.

CI, confidence interval; DoR, duration of response; INV, investigator assessment; IRC, independent review committee assessment; ITT, intent-to-treat; NE, not evaluable; PD, progressive disease.

Supplementary Table 4. Non-hematologic Grade ≥3 TEAEs in ≥3% of the Chinese subgroup (safety analysis set)

TEAEs, n (%)	Zanubrutinib (n=45)	Ibrutinib (n=43)
**Grade ≥3 TEAEs**	29 (64.4)	31 (72.1)
**Grade ≥3 TEAEs occurring in ≥3% of patients in either arm**		
Pneumonia	6 (13.3)	8 (18.6)
URTI	5 (11.1)	3 (7.0)
Hypertension	2 (4.4)	2 (4.7)
Hepatitis B	1 (2.2)	2 (4.7)
Adenocarcinoma gastric	0	2 (4.7)
Cerebral infarction	0	2 (4.7)
GGT increased	0	2 (4.7)

GGT, gamma-glutamyl transferase; TEAE, treatment-emergent adverse event; URTI, upper respiratory tract infection.

Supplementary Table 5. TEAEs leading to death in the Chinese subgroup (safety analysis set)

**TEAEs, n (%)**	**Zanubrutinib (n=45)**	**Ibrutinib (n=43)**
**Patients with ≥1 TEAE leading to death**	2 (4.4)	3 (7.0)
**Infections and infestations**	2 (4.4)	1 (2.3)
Pneumonia	1 (2.2)	1 (2.3)
Infection	1 (2.2)	0
**General disorders and administration site conditions**	0	1 (2.3)
Death	0	1 (2.3)
**Nervous system disorders**	0	1 (2.3)
Cerebral infarction	0	1 (2.3)

TEAE, treatment-emergent adverse event.

Supplementary Table 6. AESI opportunistic infections in the Chinese subgroup (safety analysis set)

Opportunistic infections, n (%)	Zanubrutinib (n=45)	Ibrutinib (n=43)
**Any opportunistic infection**	1 (2.2)	3 (7.0)
Bronchopulmonary aspergillosis	1 (2.2)	0
Herpes ophthalmic	0	1 (2.3)
Pneumonia fungal	0	1 (2.3)
Pulmonary tuberculosis	0	1 (2.3)

AESI, adverse events of special interest.

SUPPLEMENTARY FIGURES

Supplementary Figure 1. Study design diagram

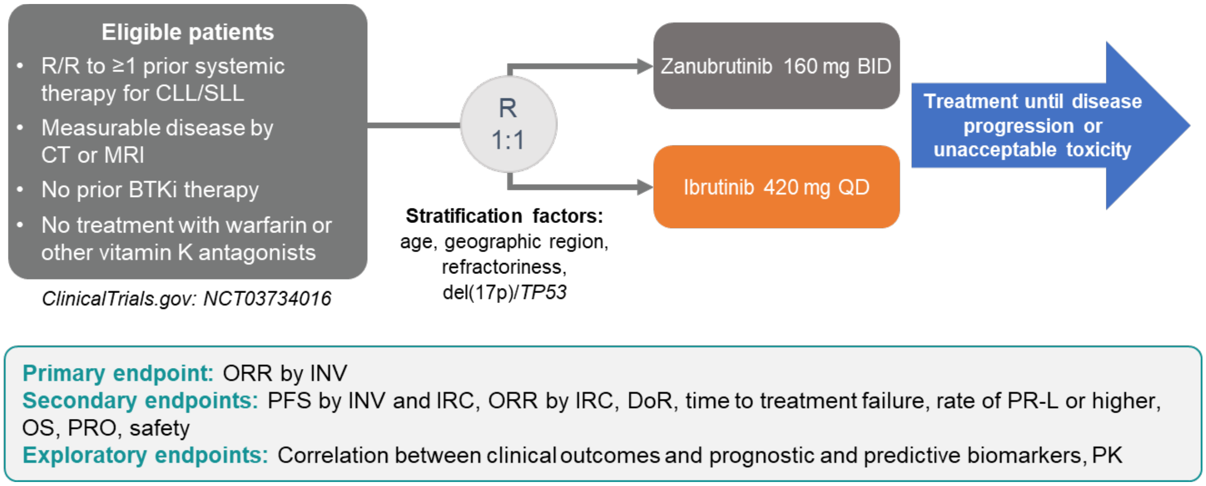

BID, twice daily; BTKi, Bruton tyrosine kinase inhibitor; CLL, chronic lymphocytic leukemia; CT, computerized tomography; del(17p), deletion in chromosome 17p; DoR, duration of response; INV, investigator assessment; IRC, independent review committee assessment; MRI, magnetic resonance imaging; ORR, overall response rate; OS, overall survival; PFS, progression-free survival; PK, pharmacokinetics; PR-L, partial response with lymphocytosis; PRO, patient-reported outcomes; QD, once daily; R, randomized; R/R, relapsed/refractory; SLL, small lymphocytic lymphoma; *TP53*, tumor protein 53.

Supplementary Figure 2. PFS by IRC in the ITT population from the Chinese subgroup

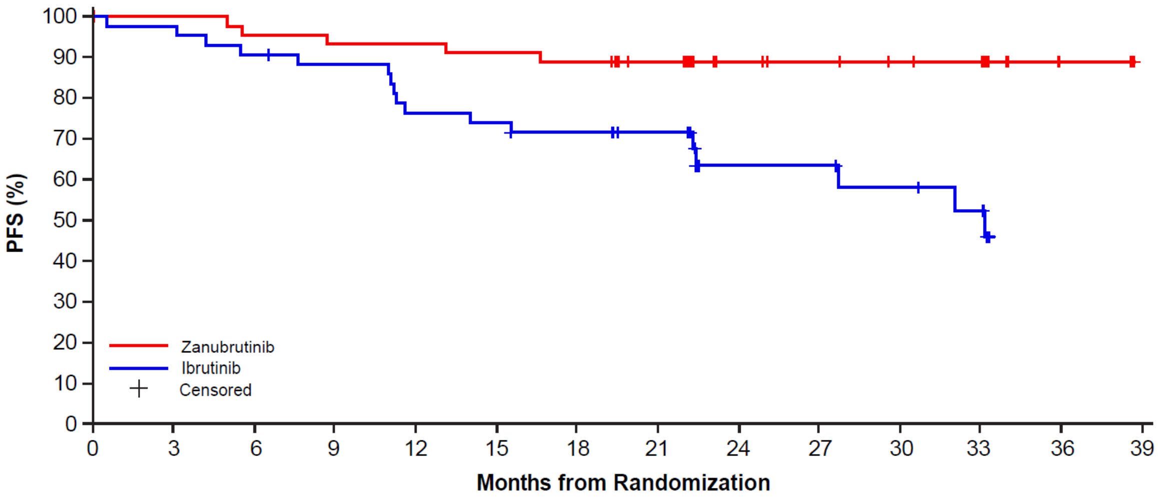

No. at Risk													
Zanubrutinib	47	45	43	42	42	41	40	32	21	19	17	16	3	0
Ibrutinib	43	42	39	37	32	31	29	25	13	13	11	9	0	

IRC, independent review committee assessment; ITT, intent-to-treat; PFS, progression-free survival.

Supplementary Figure 3. Time to treatment failure in the Chinese subgroup (ITT)

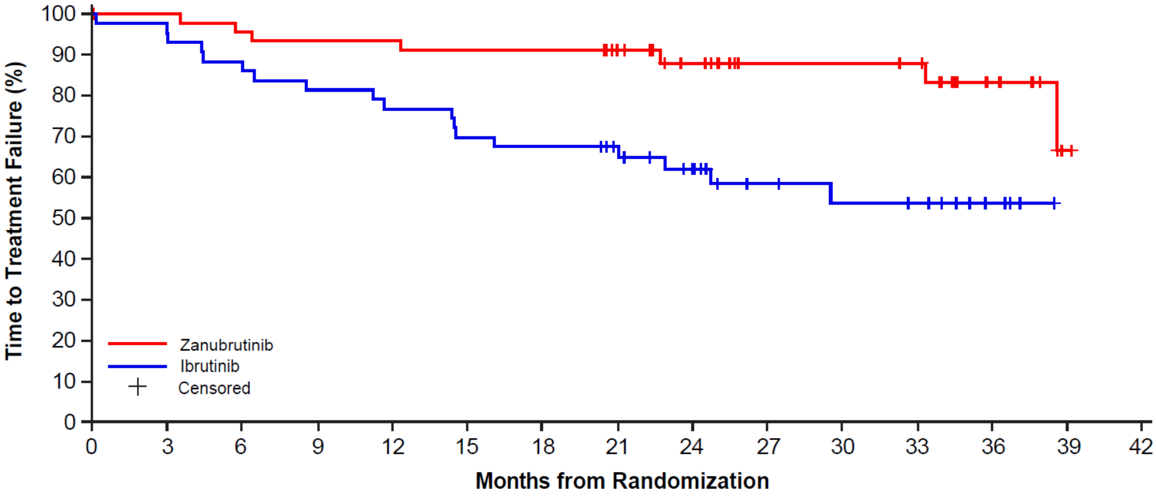

No. at Risk

Zanubrutinib	47	45	43	42	42	41	41	36	27	20	20	19	9	1	0
Ibrutinib	43	42	38	35	33	30	29	26	21	14	11	10	4	0	

ITT, intent-to-treat.

REFERENCES

1. NIH SEER Program: Cancer Stat Facts: Leukemia - Chronic Lymphocytic Leukemia (CLL). https://seer.cancer.gov/statfacts/html/clyl.html. Accessed May 24, 2023.
2. Tian Z, Liu M, Fang X, et al. (2022) Distinct Age-Related Clinical Features and Risk Assessment in Chinese With Chronic Lymphocytic Leukemia. Front Oncol 12:885150.
3. Gale RP. (2022) Chronic lymphocytic leukemia in China. Chin Med J (Engl) 135(8):883-886.
4. Ou Y, Long Y, Ji L, et al. (2022) Trends in disease burden of chronic lymphocytic leukemia at the global, regional, and national levels from 1990 to 2019, and projections until 2030: A population-based epidemiologic study. Front Oncol 12:840616.
5. Yi S, Yan Y, Jin M, et al. (2021) High incidence of MYD88 and KMT2D mutations in Chinese with chronic lymphocytic leukemia. Leukemia 35(8):2412-2415.
6. Hallek M. (2019) Chronic lymphocytic leukemia: 2020 update on diagnosis, risk stratification and treatment. Am J Hematol 94(11):1266-1287.
7. Yi S, Li Z, Zou D, et al. (2017) Intratumoral genetic heterogeneity and number of cytogenetic aberrations provide additional prognostic significance in chronic lymphocytic leukemia. Genet Med 19(2):182-191.
8. Stefaniuk P, Onyszczuk J, Szymczyk A, Podhorecka M. (2021) Therapeutic Options for Patients with TP53 Deficient Chronic Lymphocytic Leukemia: Narrative Review. Cancer Manag Res 13:1459-1476.
9. Chan TS, Lee YS, Del Giudice I, et al. (2017) Clinicopathological features and outcome of chronic lymphocytic leukaemia in Chinese patients. Oncotarget 8(15):25455-25468.
